# Interdisciplinary Radical “En-Bloc” Resection of Ewing Sarcoma of the Chest Wall and Simultaneous Chest Wall Repair Achieves Excellent Long-Term Survival in Children and Adolescents

**DOI:** 10.3389/fped.2021.661025

**Published:** 2021-03-15

**Authors:** Alireza Basharkhah, Herwig Lackner, Anna Karastaneva, Marko Bergovec, Stephan Spendel, Christoph Castellani, Erich Sorantin, Martin Benesch, Bernadette Liegl-Atzwanger, Freyja-Maria Smolle-Jüttner, Christian Urban, Michael Höllwarth, Georg Singer, Holger Till

**Affiliations:** ^1^Department of Pediatric and Adolescent Surgery, Medical University of Graz, Graz, Austria; ^2^Division of Pediatric Hematology-Oncology, Department of Pediatrics and Adolescent Medicine, Medical University of Graz, Graz, Austria; ^3^Department of Orthopedics and Trauma, Medical University of Graz, Graz, Austria; ^4^Division of Plastic, Aesthetic and Reconstructive Surgery, Department of Surgery, Medical University of Graz, Graz, Austria; ^5^Division of Pediatric Radiology, Department of Radiology, Medical University of Graz, Graz, Austria; ^6^Diagnostic and Research Center of Pathology, Medical University of Graz, Graz, Austria; ^7^Division of Thoracic and Hyperbaric Surgery, Department of Surgery, Medical University of Graz, Graz, Austria

**Keywords:** Askin tumor, Ewing sarcoma, chest wall reconstruction, tumor resection, multimodal therapy, chemotherapy

## Abstract

**Introduction:** Ewing sarcomas of the chest wall, historically known as “Askin tumors” represent highly aggressive pediatric malignancies with a reported 5-year survival ranging only between 40 and 60% in most studies. Multimodal oncological treatment according to specific Ewing sarcoma protocols and radical “en-bloc” resection with simultaneous chest wall repair are key factors for long-term survival. However, the surgical complexity depends on tumor location and volume and potential infiltrations into lung, pericardium, diaphragm, esophagus, spine and major vessels. Thus, the question arises, which surgical specialties should join their comprehensive skills when approaching a child with Ewing sarcoma of the chest wall.

**Patients and Methods:** All pediatric patients with Ewing sarcomas of the chest wall treated between 1990 and 2020 were analyzed focusing on complete resection, chest wall reconstruction, surgical complications according to Clavien-Dindo (CD) and survival. Patients received neo-adjuvant chemotherapy according to the respective Ewing sarcoma protocols. Depending on tumor location and organ infiltration, a multi-disciplinary surgical team was orchestrated to perform radical en-bloc resection and simultaneous chest wall repair.

**Results:** Thirteen consecutive patients (seven boys and six girls) were included. Median age at presentation was 10.9 years (range 2.2–21 years). Neo-adjuvant chemotherapy (*n* = 13) and irradiation (*n* = 3) achieved significant reduction of the median tumor volume (305.6 vs. 44 ml, *p* < 0.05). En-bloc resection and simultaneous chest wall reconstruction was achieved without major complications despite multi-organ involvement. Postoperatively, one patient with infiltration of the costovertebral joint and laminectomy required surgical re-intervention (CD IIIb). 11/13 patients were treated with clear resections margins (R1 resection in one patient with infiltration of the costovertebral joint and marginal resection <1 mm in one child with multiple pulmonary metastases). All patients underwent postoperative chemotherapy; irradiation was performed in four children. Two deaths occurred 18 months and 7.5 years after diagnosis, respectively. Median follow-up for the remaining patients was 8.8 years (range: 0.9–30.7 years). The 5-year survival rate was 89% and the overall survival 85%.

**Conclusion:** EWING specific oncological treatment and multi-disciplinary surgery performing radical en-bloc resections and simultaneous chest wall repair contribute to an improved survival of children with Ewing sarcoma of the chest wall.

## Introduction

Ewing sarcomas arising in the chest wall (historically known as Askin tumors) are small round cell sarcomas, molecularly defined by gene fusions involving one member of the FET family of genes and a member of the ETS family of transcription factors (1). They mainly arise from ribs, but paravertebral, sternal and scapular localizations have also been reported (2). In 1979, Askin et al. were the first ones to describe them in children, adolescents and young adults with a female preponderance (3, 4). Over the years, it became obvious that Askin tumor does not represent an separate tumor entity. In contrast, molecular studies showed identical molecular findings as seen in Ewing Sarcoma. Therefore, the terminology Askin tumor/primitive neuroectodermal tumor is not recommended by the resent WHO classification of bone and soft tissue tumors (1).

Clinically, children usually present late with a chest wall mass, chest pain or respiratory distress due to an intrathoracic mass (5). Unspecific symptoms such as fever, enlarged superficial lymph nodes, decreased general condition and nocturnal sweat may occur (6). Rarely, patients initially present with pathological fracture or metastasis related symptoms (7). Diagnostics reveal location and volume of the primary tumor as well as infiltrations e.g., into vertebral column, lung, pericardium, diaphragm or esophagus. Differential diagnoses include the group of undifferentiated small round cell sarcomas of bone and soft tissue, neuroblastoma, lymphoma, small-cell carcinoma, rhabdomyosarcoma, monophasic synovial sarcoma, and desmoplastic small round cell tumor.

Interdisciplinary and multimodal treatment of Ewing sarcoma is guided by the appropriate EWING protocols (8, 9). Aggressive treatment regimen have been shown to lead to longer relapse-free survival (6). Radical “en-bloc” resection remains crucial facing multiple challenges such as infiltrations e.g., into lung, spine, pericardium or diaphragm. Simultaneous chest wall reconstruction remains similarly challenging when covering large defects with prosthetic materials and viable tissue to prevent instability, flailing and infections. Thus, the question arises, which surgical specialties should build one team and join their comprehensive skills when approaching a child with Ewing Sarcoma of the chest wall. The present study elucidates the surgical and oncological outcome of children with Ewing Sarcoma of the chest wall treated by a multidisciplinary team of surgical specialists orchestrated according to the individual anatomical complexity.

## Patients and Methods

Following approval of the local ethics committee (EK 33-191 ex 20/21), we performed a retrospective analysis of all patients with Ewing Sarcoma of the chest wall treated at the Departments of Pediatric and Adolescent Surgery and Pediatrics and Adolescent Medicine between January 1990 and December 2020.

**Figure 1:**
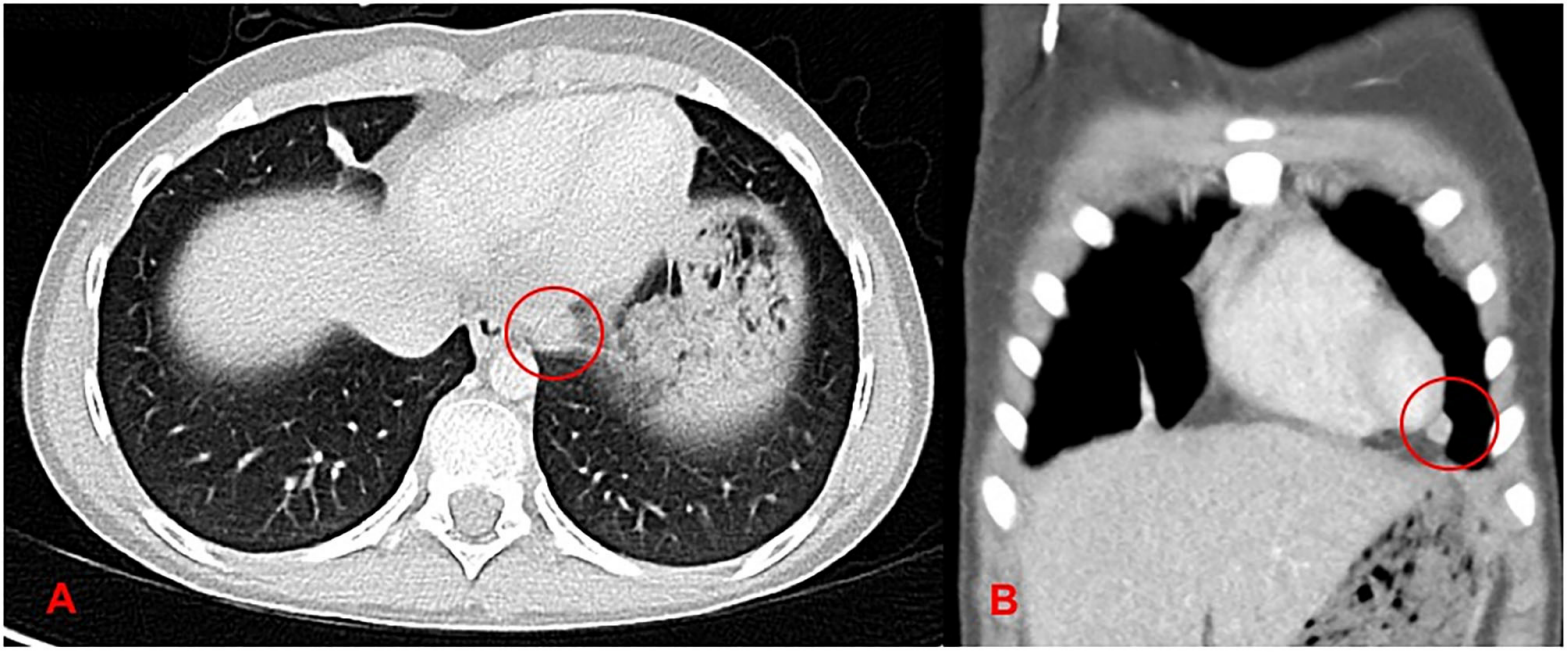
Preoperative CT revealing multiple metastases: **(A)** shows a paraesophageal metastasis (red circle) and **(B)** demonstrates a pericardial metastasis (red circle). Both were removed surgically during initial resection (Patient #13).

Patients received pediatric oncological management according to the currently valid protocol. Depending on the surgical complexity, a multidisciplinary surgical team was orchestrated to achieve radical “en-bloc” resections and simultaneous chest wall reconstructions. Pediatric surgeons or thoracic surgeons mainly performed en-bloc resections including resections of pulmonary, diaphragmatic, pericardial and esophageal infiltrations (Figure 1). They also inserted the prosthetic patches into the defect. Orthopedic surgeons took the lead for partial resection of a vertebral body and hemilaminectomy in one case with infiltration of the transverse process. Finally, plastic surgeons were responsible for covering the prosthetic patch with a muscle flap.

The diagnosis of Ewing Sarcoma of the chest wall was confirmed based on histological, immunohistochemical and molecular findings. Patient data were analyzed for presentation, tumor characteristics, oncological treatment, surgical outcome, surgical complications according to Clavien-Dindo (10), long-term follow-up and overall, progression free and relapse free survival.

## Results

From 1990 to 2020, 13 patients (*n* = 7 male, *n* = 6 female) underwent interdisciplinary multimodal therapy for Ewing Sarcoma of the chest wall. The median age of the patients at the time of presentation was 10.9 years ranging from 2.2 to 21 years.

Clinically, eight patients reported increasing thoracic pain. Three children had been treated for pneumonia. In two patients, the parents accidentally detected a thoracic swelling. One boy suffered from pneumothorax after a bout of laughter. Other unspecific symptoms included nocturnal sweating, fever, reduced general condition and collapse.

All patients underwent chest radiographs, bone scan, CT and MRI examinations revealing thoracic masses and the typical lytic bone destructions or cortical affection. Malignant pleural effusion was detected in four children, pleural seeding in three. At the time of presentation, two patients had pulmonary metastases. Detailed clinical data are presented in Table 1.

**Table 1:** Clinical data of 13 patients treated with Askin tumors between 1990 and 2020.

**ID**	**Year of diagnosis**	**Age**	**Sex**	**Primary tumor**	**Side**	**Complexity of disease**
1	1990	7	M	6th−7th	Right	Adherent to lung
2	1994	21	M	8th	Right	Pleura, adherent to diaphragm
3	2001	16.5	M	6th	Right	Malignant pleural effusion
4	2001	2.5	F	11th	Right	Adherent to diaphragm
5	2001	10.5	M	4th	Right	Adherent to lung
6	2001	18	F	9th	Left	adherent to diaphragm
7	2009	12	F	4th−6th	Right	Left lower lobe, malignant pleural effusion
8	2012	16.5	M	7th	Left	Malignant pleural effusion, tumor embolism, adherent to diaphragm and the lung lower lobe
9	2014	11	M	4th	Left	Malignant pleural effusion, adherent to the lung lower and upper lobe
10	2017	3	F	7th	Left	Adherent to vertebral body and transverse process
11	2018	8	M	3th	Right	Adherent to lung
12	2019	9	F	6th	Right	Pleura
13	2020	11.5	F	3th	Left	Pleura, left lower lobe, diaphragmatic, paraesophageal, pericardial

### Neoadjuvant Treatment

All patients underwent diagnostic biopsies. On H&E morphology the tumors were composed of uniform small round tumor cells with fine chromatin, inconspicuous nuclei and scant clear or eosinophilic cytoplasm. The individual tumor cells were evenly spaced. Immunohistochemical examination revealed a strong membranous CD99 staining in all tumors. Tumors in the differential diagnosis were excluded using a broad immunohistochemical panel of antibodies. Molecular analyses were performed in 11/13 cases. Depending on the year of diagnosis FISH, RT-PCR and Archer fusion plex sarcoma panel were performed to confirm the diagnosis of Ewing Sarcoma. One boy had an additional tri-/tetrasomy of chromosome 8.

Chemotherapy was administered in all patients, i.e., two patients in the early nineties were treated according to the CESS 86 (11) and EICESS 92 (12), four and six according to the Euro-E.W.I.N.G. 99 (8) and EWING 2008 (9) protocols, respectively. In one patient (date of diagnosis 2009) initially diagnosed as desmoplastic round cell tumor, the diagnosis was revised after the patient developed a pulmonary metastasis. She was initially treated according to CWS 2002 (13) and postoperatively according to EURO-EWINGS 99. Three patients underwent irradiation additionally to their neoadjuvant chemotherapy due to malignant pleural effusion and pleural carcinosis.

Neo-adjuvant chemotherapy and irradiation led to a statistically significant reduction of the tumor volume from a median of 305.6 ml (ranging from 32 to 814 ml) to 44 ml (ranging from 2 to 215 ml) (*p* < 0.001, Wilcoxon Test) (Table 2).

**Table 2:** Treatment of 13 patients with Askin tumors.

**ID**	**Preoperative therapy**	**Initial tumor volume (ml)**	**Tumor volume after CTX (ml)**	**Procedure performed**	**Surgical team**	**Material used for reconstruction**	**Complications CDC in 30 days**
1	CESS 86/irradiation	32	4	Resection of the dorsal part of the 6th, the entire 7th rib, the transverse process and partial resection of the lung lower lobe	PS		
2	EICESS 92/irradiation	810	215	Partial resection of the 7th−9th ribs with adherent diaphragm	TS	Corium flap plastic and m. latissimus dorsi flap at a later time point autologous transplantation of the contralateral 7th rib and m. rectus abdominis flap at a later time point	
3	EURO EWING 99	148	2	Partial resection of the 5th−7th ribs	PS, OT	Vicryl® mesh and m. latissimus dorsi flap	
4	EURO EWING 99	246.5	16	Partial resection of the 10th−12th ribs with adherent diaphragm	PS, PL	Vicryl® mesh and m. latissimus dorsi flap	
5	EURO EWING 99	813.7	15.2	Partial resection of the 3rd−5th ribs with adherent lung	PS	Goretex® patch, Vicryl® mesh	
6	EURO EWING 99	199	5.2	Partial resection of the 8th−10th ribs with adherent diaphragm	PS	Goretex® patch, Vicryl® mesh	
7	CWS 2002 pilot	216	88.6	Partial resection of the 4th−7th ribs	PS	Tutomesh® plastic and m. latissimus dorsi flap	
8	EWING 2008	175	8.8	Partial resection of the 5th−9th ribs with adherent diaphragm and the lung lower lobe	OT, TS	Gore® Dualmesh®, Prolene® Mesh m. latissimus dorsi flap	
9	EWING 2008	310	37	Partial resection of the 3rd−7th ribs and the adherent lung lower and upper lobe	PS, TS, PL	Prolene® Mesh m. latissimus dorsi flap	
10	EWING 2008	72	15	Partial resection of 6th−8th with partial vertebral body resection/hemilaminectomy, resection of the transverse process and dorsal root ganglia	PS, OT, PL	Prolene® Mesh m. latissimus dorsi flap and m. trapezius	Liquor fistula lumbar drainage
11	EWING 2008	83	5.4	Partial resection of 2nd−4th ribs and adherent lung	PS, OT, PL	Prolene® Mesh m. pectoralis flap	
12	EWING 2008/ irradiation	53.1	2	Partial resection of the 4th−6th and pleural metastases	PS, OT, PL	Prolene® Mesh m. latissimus dorsi flap	
13	EWING 2008	814	158	Partial resection of the 1st−5th ribs with metastases to pericardium, lung, diaphragm	PS, OT, PL	Prolene® Mesh m. latissimus dorsi flap	

*CTX, chemotherapy; PS, Pediatric Surgery; OT, Orthopedic Surgery; PL, Plastic Surgery; TS, Thoracic Surgery; CDC, Clavien-Dindo Classification*.

### Perioperative Course

**Figure 2:**
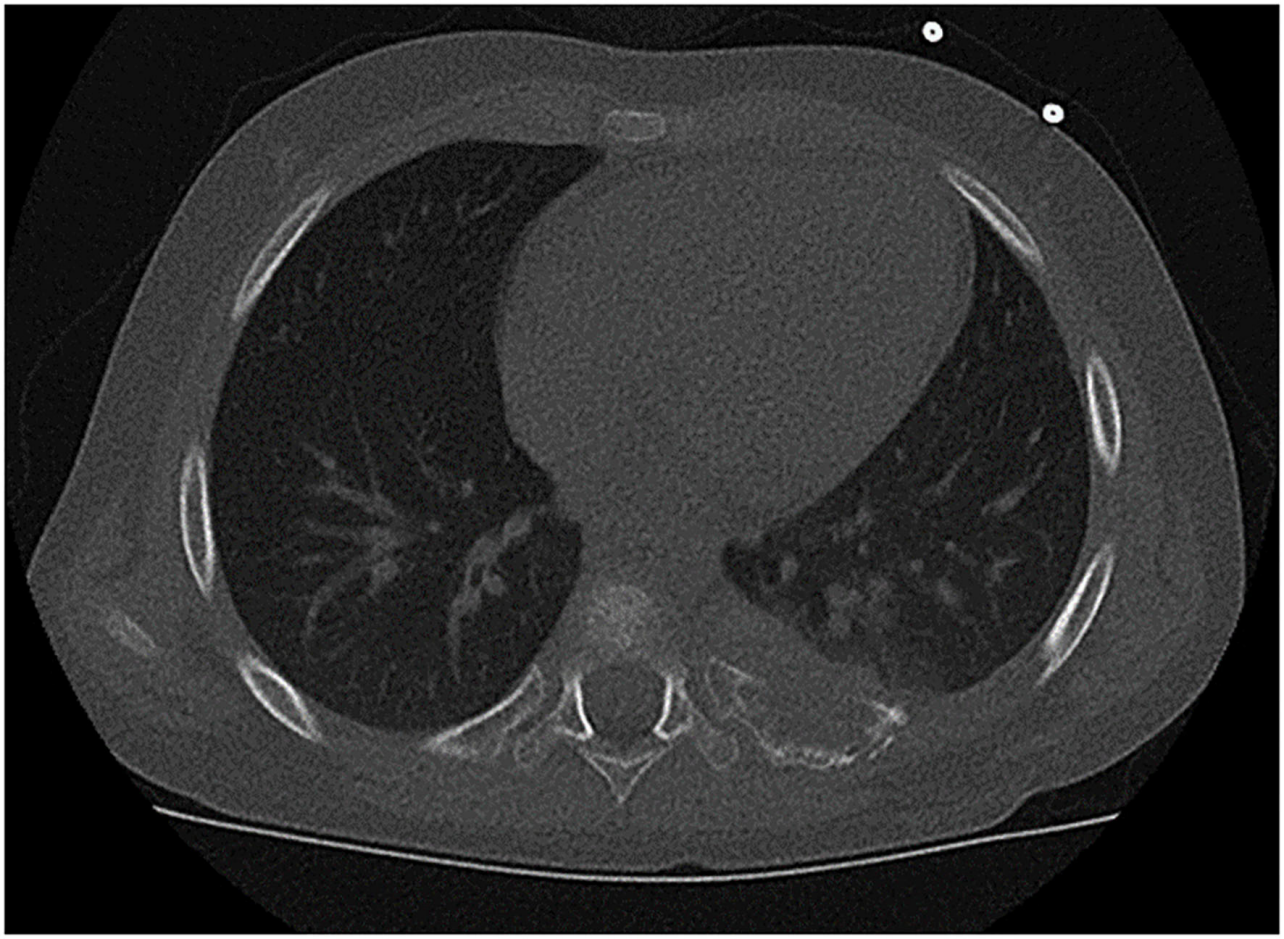
Tumor arising from the paravertebral dorsal portion of the left 7th rib with infiltration of the transverse process and vertebral body. This extent required en bloc resection of the ribs 6–8 and additional removal of the costovertebral joint and hemilaminectomy (patient #10).

En-bloc resection and simultaneous chest wall reconstruction was achieved in all cases without major complications despite multi-organ involvement. The affected rib and at least two adjacent ribs was removed in continuity with all infiltrations. Additional distant lesions were excised as well. In one case, orthopedic surgeons successfully performed partial vertebral body resection and hemilaminectomy for infiltrations into the transverse process (Figure 2). Pediatric and plastic surgeons could cover all chest wall defects with a prosthetic patch and muscle flaps. Postoperatively, the patient with infiltration of the costovertebral joint and hemi-resection of one vertebral body developed a liquor fistula requiring surgical intervention (placement of a lumbar drainage; CDC IIIb).

Table 2 summarizes detailed information concerning procedure, specialists of the team, chest wall reconstruction and short-term complications (30 days) according to CDC.

Histologically, complete resection was achieved in 11/13. R1 resection was found in one patient with infiltration of the costovertebral joint. Marginal resection of <1 mm was found in one child with multiple pulmonary metastases (Table 3).

**Table 3:** Outcome and complications of 13 patients treated with Askin tumors.

**ID**	**Local resection status**	**Grade of regression**	**Postoperative therapy (CTX/irradiation)**	**Auto-PSCTx**	**Relapses**	**Outcome years**	**Sequelae**
1	R0	1	+/–		–	30.7	Scoliosis, pulmonary restriction
2	R0	1	+/–	+	Lung/local	^†^7.5	
3	R0	1	+/+	–	–	20	
4	R0	1	+/–	–	–	19.9	
5	R0	3	+/–	–	–	19.5	
6	R0	1	+/–	–	–	19.4	
7	R0	5	+/+	+	Lung bilateral	†1.5	
8	R0	1	+/refused	+	–	8.8	
9	R0	1	+/–	+	–	7	Pulmonary restriction
10	R1	5	+/+	–	Local/cervical	3.4	Scoliosis
11	R0	1	+/–	–	–	2.9	
12	R0	1	+/–	+	–	1.2	
13	Marginal	5	+/+	+	–	0.9	

*CTX, chemotherapy; PSCTx, peripheral stem cell transplantation*.

† *deceased*.

Histologic evaluation of the tumor resection specimen after multimodal treatment revealed no viable appearing tumor cells in 9 cases therefore classifying them as Salzer-Kuntschik I grade of regression. In one case the regression grade was III (<10% vital tumor cells) and in the three remaining patients there were more than 50% vital tumor cell (regression grade V) (14).

All patients underwent adjuvant chemotherapy. Additional postoperative irradiation was administered in four cases, due to the above-mentioned marginal resection (*n* = 2), malignant pleural effusion (*n* = 1) and pulmonary metastases (*n* = 1). One patient with a histological confirmed invasion of tumor into a small pulmonary artery and malignant pleural effusion (patient #8) refused the advised radiotherapy.

### Long-Term Outcome and Sequelae

Overall survival rate with multimodal therapy is 85% at a median follow-up of 8.8 years (ranging from 0.9 to 30.7 years). The 5-year survival rate is 89%. The median progression free survival was 7 years (range: 0.4–30.7 years). While one patient is still under the primary therapy and one patient passed away during primary therapy, the median relapse free survival in the remaining 11 patients was 8 years (0.4–30 years).

Eight out of the 13 patients had an uneventful oncological follow-up without signs of relapse or sequelae (Table 3). Patient #10, who required partial vertebral body resection developed postoperative scoliosis. A local relapse 2 years later necessitated radiotherapy. Three years after the primary diagnosis, a metastasis was found in the cervical vertebral column (C2) and she is currently under chemotherapy and radiotherapy. Two further patients had postoperative complications: Patient #1 developed postoperative scoliosis and pulmonary restriction with a tumor free interval of 30 years and patient #9 suffers from pulmonary restriction at a tumor free interval of 6.5 years.

Two patients died. In Patient #2 pulmonary metastasis occurred 5 years after the initial diagnosis. The metastasis was resected and chemotherapy including high-dose chemotherapy with autologous stem cell support was administered. However, two further relapses occurred, and he passed away 7.5 years after the initial diagnosis.

Patient #7 who initially presented with pleural effusion and pulmonary metastasis in the contralateral lower lobe developed six bilateral lung metastases later. Despite resection via sternotomy, chemotherapy and radiation the girl suffered from multiple recurrent metastases in both lungs and died 18 months after the first diagnosis.

## Discussion

With an aggressive multimodal interdisciplinary team approach with “targeted” surgical specialists we achieved an 85% overall survival rate at a median follow-up of 8.8 years and a 5-year survival rate of 89%. Two of the 13 patients treated during the last 30 years died 18 months and 7.5 years after diagnosis, respectively. Two patients are still under therapy.

Ewing sarcoma of the chest wall usually have a poor prognosis. Triarico et al. have reported a series of 9 patients with “Askin tumors,” and their 5-year survival was 60% at a median follow-up of 53.1 months (15). Five-year survival of patients with localized extra-skeletal Ewing sarcomas has been described with 69.7% (16). Additionally, the overall survival rate after a median follow-up of 28 months was 45% in a study by Laskar (17).

Poor prognosis has been associated with tumor diameters > 5 cm, LDH levels > 240 U/l and late stage diagnosis (4). In another study, patients with poor histological response to chemotherapy and positive surgical margins had significantly worse event-free survival (18). Laskar et al. have shown that age at diagnosis higher than 18 years, poor response to induction chemotherapy, and presence of pleural effusion are indicators of inferior survival (17).

Cornerstones of current treatment protocols include neo-adjuvant chemotherapy, radical “en-bloc” resection and chest wall reconstruction as well as adjuvant chemotherapy/radiotherapy (19, 20).

Neo-adjuvant chemotherapy has been shown to prolong survival rates (21, 22). This may be attributed to the formation of a pseudo-capsule, which reduces the risk of intraoperative tumor rupture and tumor cell dissemination (21, 23, 24). By shrinking tumor volume it decreases vascularity and vulnerability and improves feasibility of complete removal with negative microscopic margins (4, 25–27). In our study, we confirmed the efficacy of neo-adjuvant chemotherapy with a significantly reduced tumor volume following preoperative chemotherapy (Table 2). Moreover, Demir et al. reported that neo-adjuvant chemotherapy significantly increased the complete resection rate with 5-year survival rates with or without neo-adjuvant therapy of 77 and 37%, respectively (28).

Following neo-adjuvant chemotherapy, complete “en-bloc” resection of the primary lesion and simultaneous reconstruction of the defect is mandatory. Radical surgical resection is associated with better survival; patients with complete resection have a higher 5-year survival rate compared to patients who had an incomplete resection (28). The surgical complexity primarily depends not only on tumor location and volume, but also on the extent of infiltrations into lung, pericardium, diaphragm, esophagus or spine. Thus, the question arises, which surgical specialists should build one team and join their comprehensive skills when approaching a child with Ewing sarcoma of the chest wall. In the literature, there are some reports about the team approach (6, 15, 29), but the studies report lower survival rates than those achieved in ours. We orchestrated the team according to the surgical complexity and organ involvement, targeting “en-bloc” resection with a preferably macroscopic safety margin of 1 cm. This strategy is technically more demanding when the first and second rib have to be removed. Care should be taken to avoid injury of subclavian vessels and brachial plexus.

Simultaneously, all distant metastases within the ipsilateral thoracic cavity must be managed requiring pulmonary, cardiac, esophageal and diaphragmatic expertise. In our setting pediatric surgeons or thoracic surgeons faced these challenges successfully with minor complications. Finally, simultaneous repair of the chest wall defect requires distinct surgical finesse. A variety of materials is available to cover the defect. This includes synthetic meshes, bioprosthetic materials, stainless-steel bars, osseo-integrated titanium systems, autografts, homografts or porcine or bovine xenografts. In 2020, Smelt et al. described the successful application of personalized three-dimensional (3D)-printed chest wall prostheses made of methylmetacrylate covered by expanded polytetrafluoroethylene for patients undergoing chest wall resection and reconstruction (30). However, due to residual growth and development, the choice of the optimal material is a major consideration in the pediatric and adolescent population. The material should be malleable enough to conform to the shape of the chest wall, rigid enough to prevent paradoxical motion and protective for the intrathoracal organs. Additionally, it should be durable, non-allergenic, non-toxic, biologically inert and radiolucent (31, 32). One advantage of synthetic meshes used in our patients is their permeability, which minimizes the amount of postoperative pleural effusion. The mesh is sutured tautly to the surrounding tissue allowing ingrowth of connective tissue and incorporation of the mesh into the body (33). Most centers also insert a prosthetic patch into the skeletal defect and cover it with viable tissue like a muscular flap. This dual layer technique offers the advantage that well-vascularized tissue facilitates integration of the underlying material into the surrounding tissue thereby minimizing the risk of infections (34). In our series no infection of the patches or flaps occurred.

Nevertheless, long-term sequelae such as scoliosis cannot always be prevented. In our series, two of the 13 patients developed scoliosis. In a recently published report, Marqués et al. describe the successful operative correction of four pediatric patients with scoliosis secondary to extensive chest resections due to Askin tumors (35). Moreover, female patients should be also observed regarding mammary sequelae. However, due to the rarity of the disease there is no real recommendation for mammary reconstruction in affected female patients.

Local relapse represents a major negative factor for mortality (36). Christiansen et al. described a series of 8 patients, in which 4 out of 5 patients with relapse died (6). In our series, we found similar mortality rates as two out of three patients with relapses died and the third one is still under treatment. In 2013, we have already published our experience with seven patients with Ewing sarcoma of the chest wall. We were able to achieve a 5-year survival rate of 86% and an overall survival of 71% (22). By adding another 6 patients in the current report, we support this relatively high survival rate.

Postoperative irradiation was applied in four of the patients. In two of them due to viable tumor cells with Salzer-Kuntschik grade V regression and marginal resection, in one boy with a malignant pleural effusion and in one girl with multiple pulmonary metastases. Patient #8 refused the recommended postoperative radiotherapy. Radiotherapy should only be reserved for individual patients with unfavorable non metastatic tumors or for patients with a high risk of recurrence, such as in an incomplete resection or viable tumor (26, 37), due to the late effects related to radiotherapy such as chest wall deformities, pulmonary fibrosis and risk of secondary malignancies (19).

Limitations of the present study include the relatively low number of patients and the short observation period of four patients (patients 10–13). However, due to the rarity of Ewing sarcomas in general and the location chest wall in particular large series in children and adolescents concentrating on the surgical approach are scarce in the literature.

In conclusion, Ewing sarcoma specific oncological (neo)adjuvant treatment and multi-disciplinary surgery performing radical en-bloc resections and simultaneous chest wall repair contribute to an improved long-time survival of children and adolescents with Ewing sarcoma of the chest wall.

## Data Availability Statement

The raw data supporting the conclusions of this article will be made available by the authors, without undue reservation.

## Ethics Statement

The studies involving human participants were reviewed and approved by Ethics Committee of the Medical University of Graz. Written informed consent from the participants' legal guardian/next of kin was not required to participate in this study in accordance with the national legislation and the institutional requirements.

## Author Contributions

AB, CC, GS, and HT analyzed the data, wrote the manuscript and performed the statistics. AB, HL, AK, MBer, SS, CC, ES, MBen, BL-A, F-MS-J, CU, MH, and HT operated, treated and examined the patients and critically reviewed the manuscript. All authors contributed to the article and approved the submitted version.

## Conflict of Interest

The authors declare that the research was conducted in the absence of any commercial or financial relationships that could be construed as a potential conflict of interest.
